# Enhancing sleep stage classification with ballistocardiogram signals: feature selection using attention mechanism and XGBoost

**DOI:** 10.3389/fpubh.2025.1608725

**Published:** 2025-07-28

**Authors:** Chao Luo, Banteng Liu, Jiayu Chai, Zhijian Teng

**Affiliations:** ^1^School of Information Engineering, Huzhou University, Huzhou, China; ^2^Key Laboratory of Artificial Organs, Computational Medicine in Zhejiang Province, College of Information Science and Technology, Zhejiang Shuren University, Hangzhou, China

**Keywords:** sleep staging, ballistocardiogram (BCG), heart rate variability (HRV), respiratory rate variability (RRV), XGBoost, feature selection

## Abstract

Traditional sleep staging using contact sensors may compromise data validity. This study proposes a non-contact ballistocardiogram (BCG)-based method to improve both accuracy and comfort in sleep monitoring. BCG signals were processed using continuous wavelet transform and low-pass filtering to extract heart rate variability (HRV) and respiratory rate variability (RRV). A novel feature selection model integrating attention mechanisms with XGBoost was developed, where attention weights are used to prioritize features before iterative refinement by XGBoost. Evaluated on 10,201 sleep segments, the Fast-ABC Boost model achieved an accuracy of 89.85%, along with superior precision, recall, F1-score, and Kappa values compared to conventional methods. The attention-XGBoost fusion effectively mitigates interference from noisy and redundant features while optimizing feature relevance, demonstrating robust adaptability to the complexity of sleep signals. This innovation advances the accuracy non-contact sleep staging, enabling practical applications in home healthcare and personalized sleep management, while improving user comfort.

## 1 Introduction

**Figure 1:**
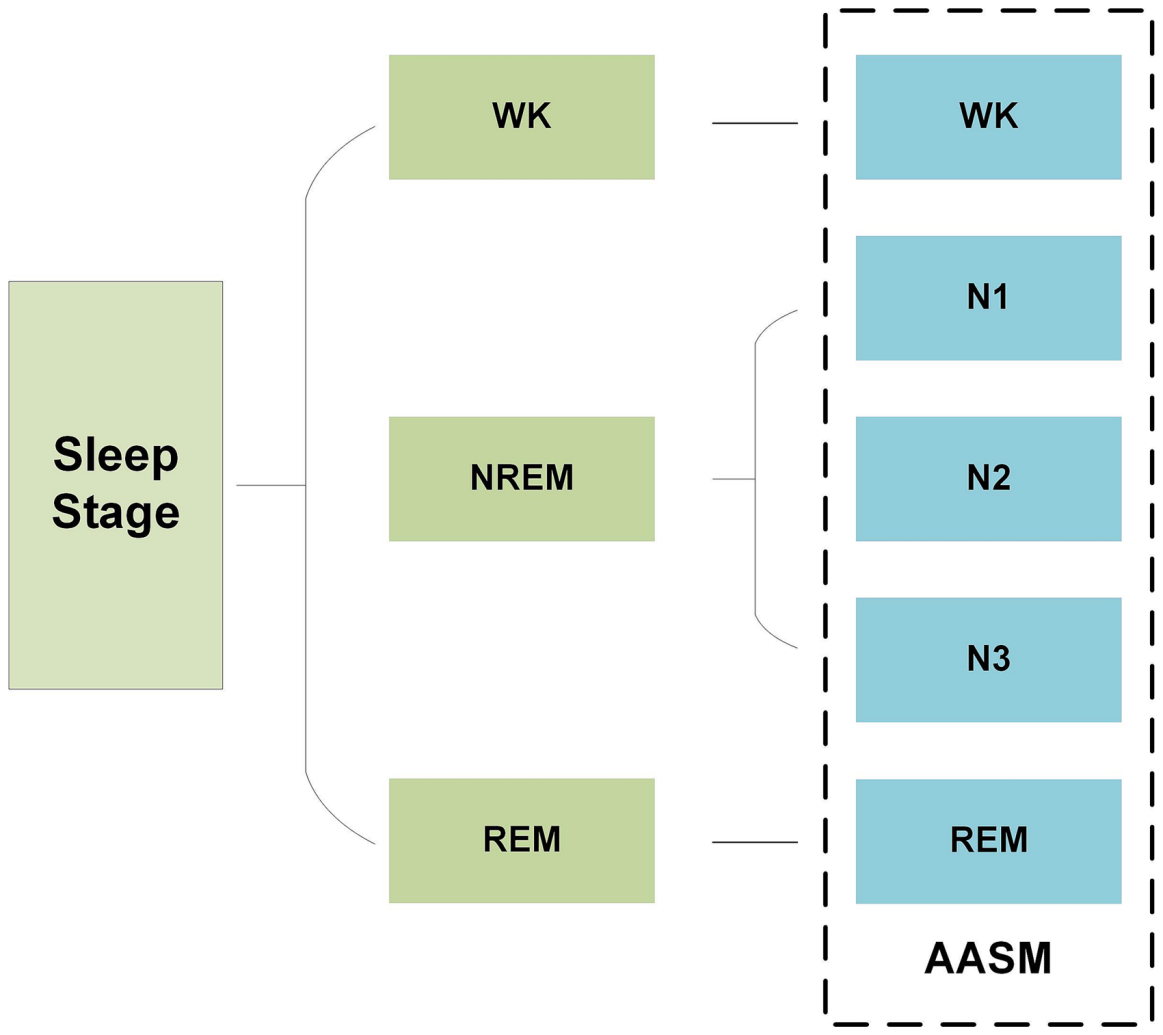
Sleep staging guidelines released by AASM.

In recent years, as the pace of modern life has accelerated, sleep patterns have undergone significant changes, leading to a continuously rise in the incidence of sleep-related disorders (1). In clinical practice, physiological indicators from different sleep stages are widely used to diagnose and monitor sleep disorders, assess sleep quality, and provide crucial support for developing of scientific treatment plans and intervention strategies. Accurately distinguishing and identifying each sleep stage is not only essential for gaining a deeper understanding of sleep mechanisms, but also a key aspect of improving the management of sleep disorder and enhancing individual health. In 2007, the American Academy of Sleep Medicine (AASM) released the latest sleep staging guidelines, which divide sleep into five stages: wakefulness (Wakefulness, W), rapid eye movement (Rapid Eye Movement, REM) sleep, and three non-rapid eye movement (Non-Rapid Eye Movement, NREM) stages (N1, N2, N3) (2), as shown in Figure 1. Based on this standard, current sleep research typically divides the entire sleep process into 30-s intervals, labeling each interval with the corresponding sleep stage to facilitate subsequent analysis.

Traditional studies have shown that heart rate variability (HRV) features play a crucial role in sleep stage monitoring, and these HRV features are typically extracted from electrocardiogram (ECG) signals. HRV analysis from ECG has been used to study sleep, revealing that the low-frequency HRV power and heart rate are lower during deep sleep compared to REM sleep (3). Following this successful study, other researchers proposed sleep stage estimation algorithms based on ECG-derived HRV (4, 5). However, these methods require multiple electrodes to be directly attached to the patient’s skin during measurement (6–8), which can cause inconvenience in daily life. From the perspective of sleep research, long-term non-invasive HRV monitoring holds significant value.

Ballistocardiogram (BCG) signals reflect cardiac activity. Unlike ECG, BCG can be measured non-contact using pressure sensors, without the need for direct electrode contact with the body, and the equipment is low-cost. Studies (9–11) have demonstrated good consistency between BCG and ECG signals in HRV analysis through comprehensive statistical analysis. Therefore, using BCG signals for high-accuracy sleep staging offers important research value.

Currently, various types of sensors are used to collect BCG signals, such as chairs (12), weight scales (13), and sheet sensors (14). Among them, sheet sensors (15) have emerged as the ideal choice for sleep studies, as they can be placed directly under the mattress to continuously monitor heart rate during sleep.

**Figure 2:**
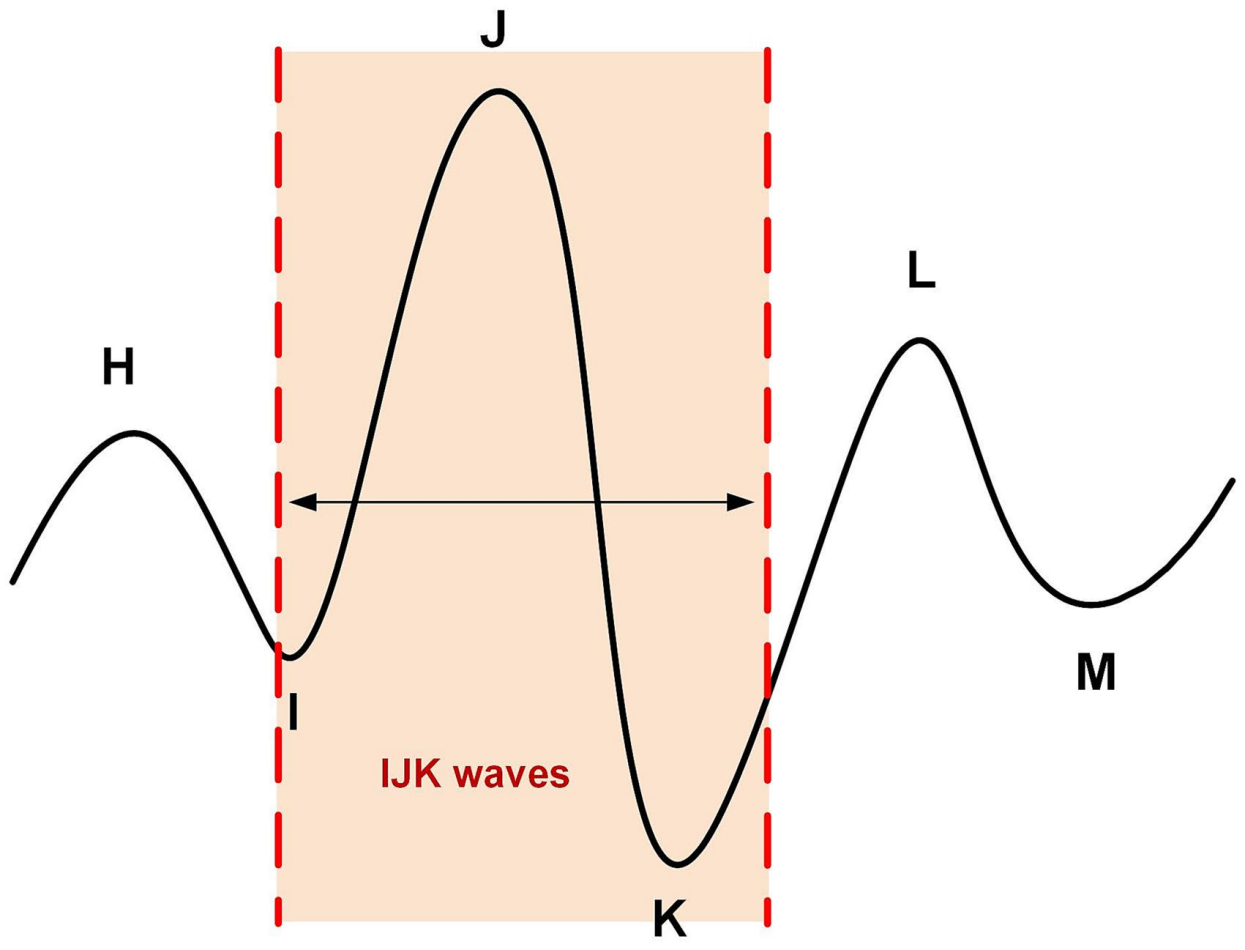
Schematic diagram of BCG signal waveform.

A typical BCG signal contains multiple peaks, such as H, I, J, K, L, M, etc. (16, 17), as shown in Figure 2. The I-J-K waves in the BCG waveform are the main components and are typically used as features for detecting heartbeats.

BCG signal processing aims to extract heartbeat and respiratory information, which are crucial for sleep staging. Digital filtering is a widely used, efficient, and reliable method. For instance, Alvarado-Serrano et al. (18) employed continuous spline wavelet transform to reconstruct heartbeat signals and estimated heart rate using an adaptive threshold method. Thirion et al. (19) applied a moving average filter and a Butterworth IIR filter to extract heart rate signals. Liu et al. (20) optimized the parameters of variational mode decomposition (VMD) to analyze heart rate signals from fiber Bragg grating sensors. Wu et al. (21) designed an adaptive soft filter with convolution kernels of varying sizes to extract heartbeat waveforms. Sadek et al. (22) compared MODWT, CWT, and template matching for heart rate detection, finding that CWT with a Gaussian function performed best, while MODWT was the most time-efficient.

Most studies utilize heart rate variability (HRV) features extracted from BCG signals for sleep staging. Suliman et al. (23) and Mitsukura et al. (24) employed template matching and SVM models for five-stage classification based on HRV features. Liu et al. (25) analyzed heartbeat intervals and HRV features for both two-stage and four-stage sleep staging, while Yoshihi et al. (26) incorporated body movement data to improve stage estimation. Meanwhile, BCG signals also provide respiratory information (27), which can be used to extract respiratory rate variability (RRV) features. Yi et al. (28) and Wu et al. (29) integrated HRV and RRV features for multi-stage sleep classification using machine learning models, while Ahmed et al. (30) further incorporated motion data from BCG signals for sleep–wake classification using deep learning techniques. The combination of HRV and RRV features has demonstrated potential in enhancing sleep staging accuracy.

Despite significant progress in sleep staging based on BCG signals, several challenges remain. BCG signals are highly susceptible to individual differences and external noise due to their dependence on body movements and cardiac mechanical activities. In high-noise environments, signal contamination or loss can hinder heartbeat extraction. Additionally, real-world BCG datasets often suffer from severe class imbalance, with the N2 stage typically accounting for more than 40%, while the N1 stage represents less than 5%.

**Figure 3:**
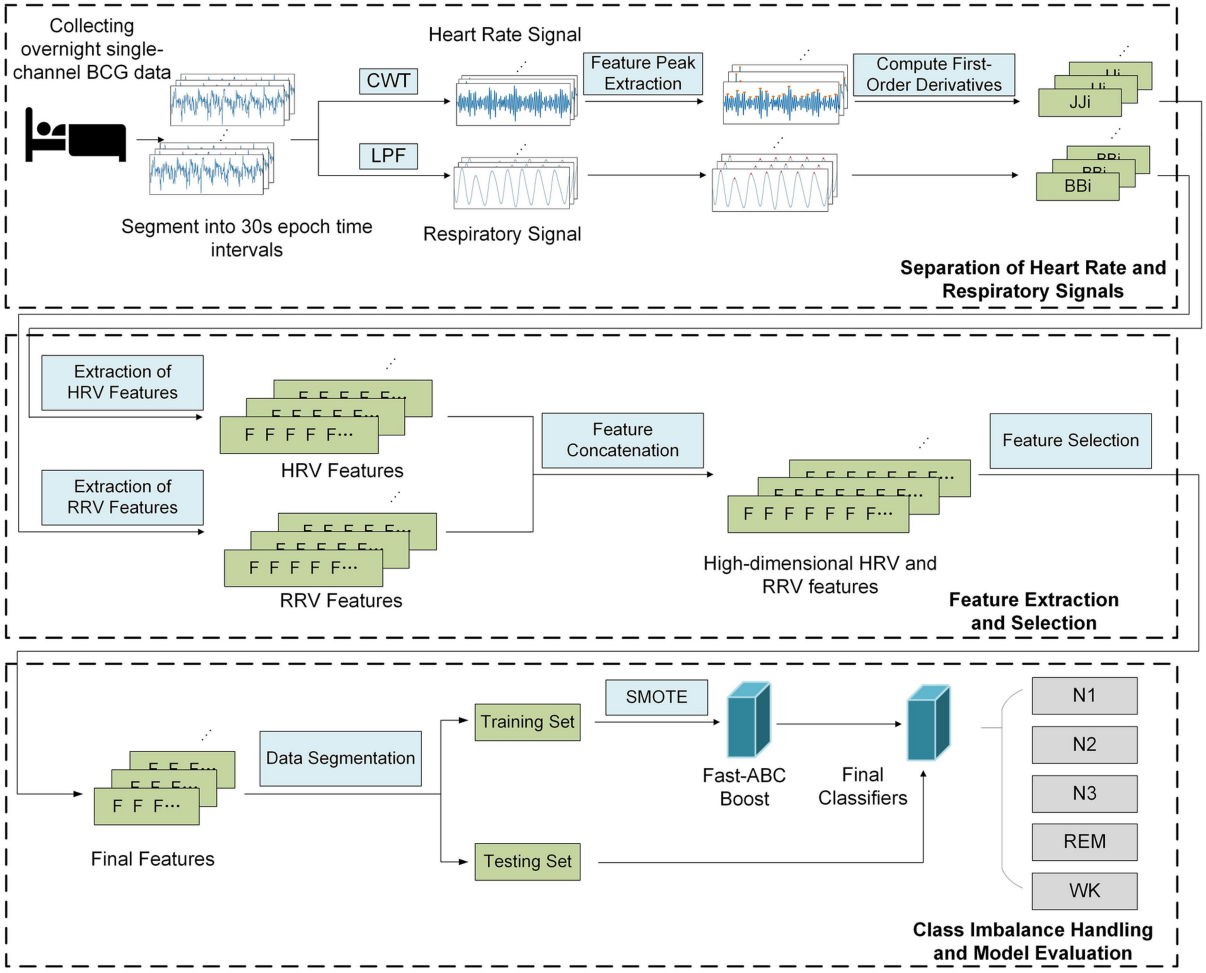
Diagram of sleep staging method based on BCG signal.

To address above challenges, this study proposes a sleep staging method based on BCG signals and the Fast-ABC Boost model, which prioritizes misclassified minority classes to enhance overall classification performance. Furthermore, an attention mechanism combined with the XGBoost algorithm is introduced for feature selection, enhancing robustness against noise and imbalanced data. Experimental validation on a BCG-based sleep staging dataset demonstrates the superiority of this approach compared to traditional feature selection techniques. As shown in Figure 3, the proposed framework consists of following main components:

1. Separation of Heart Rate and Respiratory Signals: The first step involves using multi-scale continuous wavelet transform (CWT) and a low-pass Butterworth filter to separate the heart rate and respiratory signals from the original BCG signals. Next, the signal is divided into 30-s intervals, and the positions of the characteristic peaks are extracted to calculate the first-order difference, thus forming the JJ_i_ and BB_i_ sequences.
2. Feature Extraction and Selection: Based on the JJ_i_ and BB_i_ sequences, multi-dimensional HRV and RRV features are extracted through analysis and calculation. Long-term features are then extracted using extended time windows. An attention mechanism, combined with the XGBoost algorithm, is applied to mitigate the impact of redundant and noisy features, while selecting the most critical features for sleep staging.
3. Class Imbalance Handling and Model Evaluation: The BCG sleep data were collected from 10 subjects, with full-night recordings from each. The data were combined into a single dataset and randomly split into training and testing sets. To address class imbalance, SMOTE (Synthetic Minority Over-sampling Technique) was applied to augment the minority class in the training set. The Fast-ABC Boost model was then used to evaluate performance on the real-world sleep data.

## 2 Methods

### 2.1 Data preprocessing

#### 2.1.1 Heart rate signal processing

**Figure 4:**
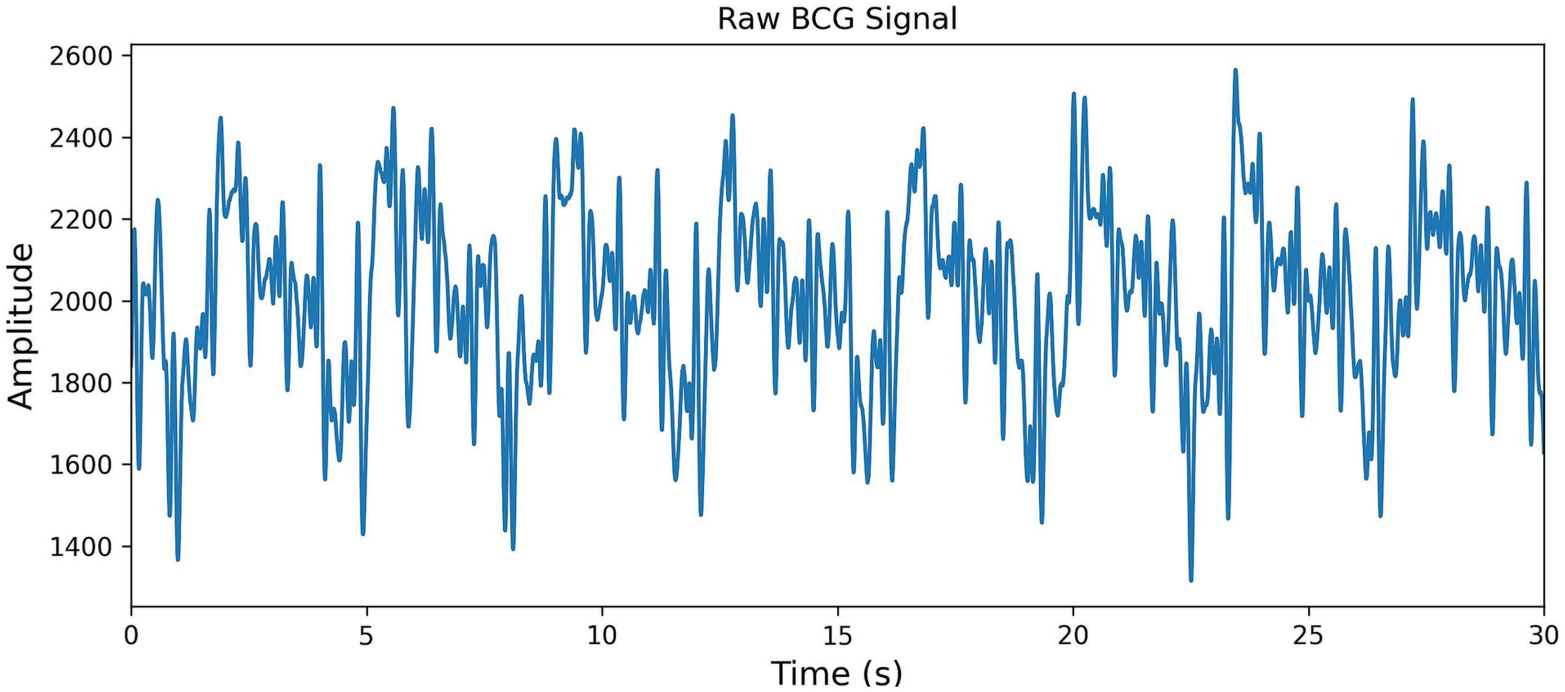
Visualization of raw BCG signal.

BCG signals (as shown in Figure 4) are highly sensitive to both external and internal factors, and their characteristics are significantly influenced by individual differences and temporal variations. Physiological factors such as body constitution, weight, and cardiac structure of different individuals can affect the intensity and morphology of BCG signals. Additionally, even within a single individual, over time, BCG signals are influenced by dynamic factors such as breathing patterns, posture changes, and position adjustments, which can lead to increased signal volatility, making it challenging to construct a robust temporal representation model. Furthermore, BCG signals are highly susceptible to environmental noise, device vibrations, and external interference during the acquisition process, which can significantly deteriorate the quality of the data (31). The analysis of heart rate signals typically relies on the accurate detection of the J-peak within the heartbeat cycle. By obtaining the J-peak location and calculating the first-order difference, the heartbeat interval sequence can be obtained for further HRV analysis.

To effectively extract heart rate event information from the BCG signal and adapt flexibly to its multi-frequency characteristics, this study uses a multi-scale continuous wavelet transform (CWT) filtering method. By generating CWT decompositions at different scales, the scale with the largest combined energy and variance is selected for feature extraction.

**Figure 5:**
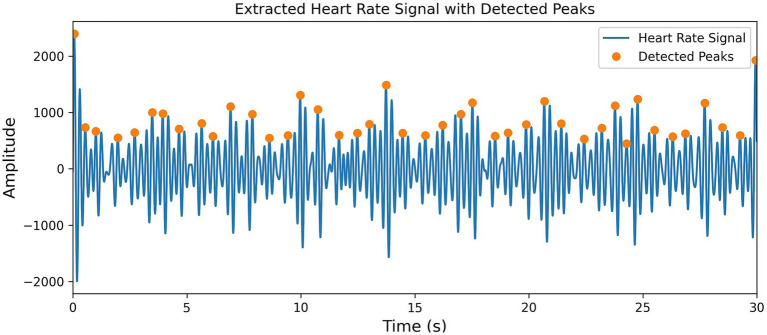
Visualization of peak extraction from heart rate signal features.

To precisely locate the J-peak, a reasonable heart rate peak detection mechanism is set up. First, the height of the smallest peak is set to be greater than the average value of the positive portion, which effectively filters out low-amplitude interference signals. Then, the significance of the peak is set as 0.3 times the standard deviation, which filters out insignificant peaks and ensures that the detected peaks are more reliable. Next, the peak interval is set to be between 0.4 and 1.4 s to capture transient heart rate fluctuations caused by noise or interference, avoiding the omission of critical heart rate information. Finally, the position of the J-peak is extracted from the heart rate signal. The results are shown in Figure 5.

To eliminate random errors caused by electrode contact issues and abnormal pulsations, an outlier handling method was designed. First, the first-order difference of all J-peak positions within a 30-s segment is calculated to generate the JJ_i_ sequence, and the average value of the sequence is computed. Then, outliers that are smaller than 0.3 times the average value and larger than 1.5 times the average value are removed and replaced with the average value of the sequence.

#### 2.1.2 Respiratory signal processing

**Figure 6:**
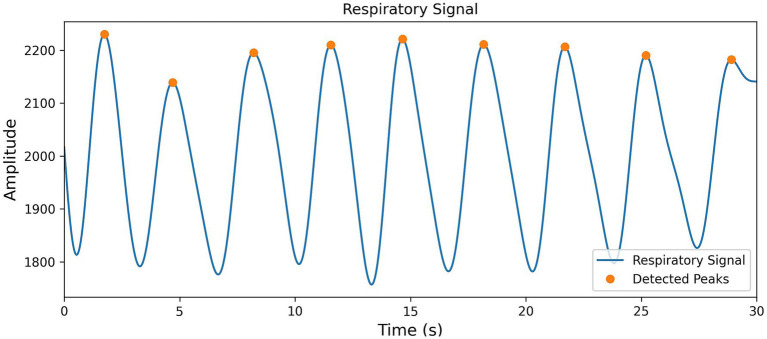
Visualization of peak extraction from respiratory signal features.

To effectively extract respiratory event information from the BCG signals, this study employs a Butterworth low-pass filter, with a cutoff frequency set at 0.5 Hz and using bidirectional filtering to effectively separate the respiratory signals from the BCG data. Then, by directly setting the distance threshold to 2 s, peak points are successfully extracted (the results are shown in Figure 6), and the first-order difference of all peak positions is calculated, forming the BB_i_ sequence.

### 2.2 Feature extraction and feature selection

#### 2.2.1 Feature extraction method based on multi-scale time window

This study adopts a multi-scale time window method to extract time-domain features at different time scales and explores the potential relationship between these features and sleep stages. By extracting time-domain features from longer time-scale windows, the long-term trends, state transitions, and physiological changes in the sleep signal can be analyzed more comprehensively. This approach helps capture the dynamic characteristics of sleep cycles, thereby improving the accuracy and robustness of sleep stage recognition. Therefore, this study concatenates each 30-s time segment with the subsequent 1, 3, 9, and 19 segments (representing 1-, 2-, 5-, and 10-min time series, respectively), from which time-domain features are extracted. This multi-scale time-domain feature extraction method aims to reveal the temporal patterns of sleep signals at different time scales, providing richer information for more precise classification of sleep stages.

#### 2.2.2 Attention mechanism for weighted feature selection

The attention mechanism is a key concept in the field of deep learning, inspired by the way biological systems in humans focus on important features when processing large amounts of information. By emulating this mechanism, models can better focus on task-relevant information when handling complex data (32). This allows the model to dynamically prioritize important features and reducing reliance on irrelevant ones.

In this study, multiple fully connected layers (Dense Layers) are used to learn more complex feature mappings, assigning weight values to each feature. BatchNormalization is added after each Dense layer to normalize activations, helping to improve training speed and stability. A Dropout layer is then included to reduce overfitting. Finally, a sigmoid activation function is used to ensure that the weights are between 0 and 1, reflecting the relative importance of each feature in the model. Features with higher weights receive more attention, while features with lower weights have less influence. This weighting mechanism allows the subsequent feature selection model to filter features more accurately, focusing on learning the most important features for the task and avoiding interference from noisy features during model training.

#### 2.2.3 XGBoost-based feature selection for high-dimensional HRV and RRV data

High-dimensional HRV and RRV features can be extracted from the JJ_i_ and BBi sequences of 30-s and longer time segments through mathematical analysis. However, these features often contain a significant amount of noise and redundancy. On the one hand, certain individual features contribute little to model accuracy and may even have a negative impact on performance, and are thus considered noise features. On the other hand, different physiological features may exhibit similar variation trends within the same sleep stage, leading to a certain degree of redundancy among features. Therefore, this study adopts the XGBoost algorithm for feature selection (33). The core idea of XGBoost is to optimize the model by minimizing the objective function, which consists of a loss function and a regularization term, as defined in Equation (1):


(1) $$\mathrm{Obj}(\theta )=\sum_{i=1}^{n}L(y_{i},{\hat{y}}_{i})+\sum_{k=1}^{K}\Omega (f_{k})$$

where $L(y_{i},{\hat{y}}_{i})$ is the loss function, representing the error between the true value $y_{i}$ and the predicted value ${\hat{y}}_{i}$ ; $\Omega (f_{k})$ is the regularization term used to control the model’s complexity and prevent overfitting. In the optimization process, XGBoost approximates the loss function through a second-order Taylor expansion, as defined in Equation (2):


(2) $$\mathrm{Obj}(\theta )\approx \sum_{i=1}^{n}[g_{i}f(x_{i})+\frac{1}{2}h_{i}f{\left(x_{i}\right)}^{2}]+\Omega (f)$$

where $g_{i}$ and $h_{i}$ are the first-order and second-order derivatives, representing the gradient and curvature, respectively, and they help the model converge more quickly. The XGBoost algorithm calculates the weight of each feature.

**Figure 7:**
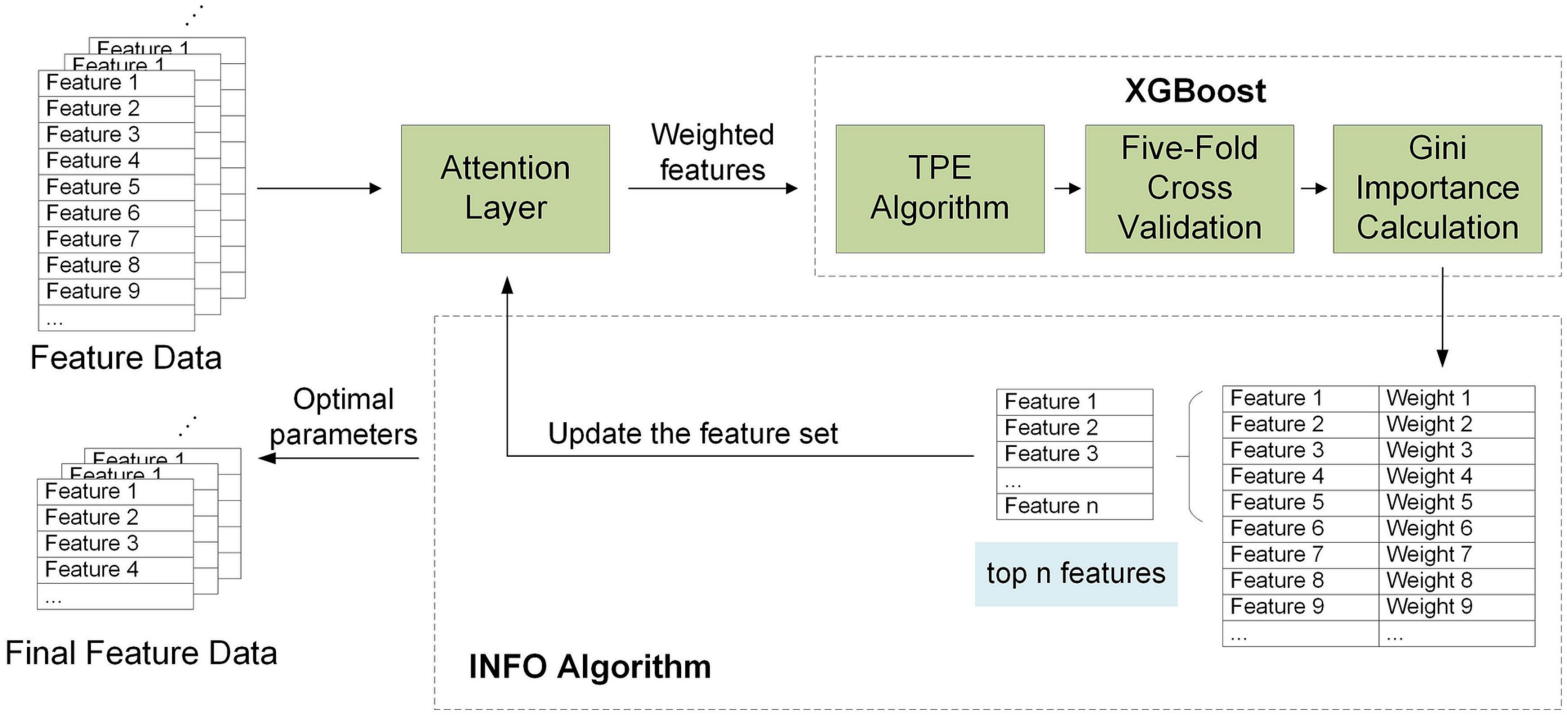
Architecture diagram of a feature selection model based on attention mechanism combined with XGBoost.

To ensure that the selected feature set is both representative and effectively captures important information related to the research objective, this study first applies the attention mechanism for feature weighting. The weighted features are then input into the XGBoost algorithm for feature selection. After obtaining the weight of each feature, the features are sorted by their weights, and those whose weight proportions exceed a certain threshold are selected. Next, a dynamic feature set update strategy is employed, where the selected features are re-input into the algorithm for further iterative calculation, gradually refining the feature set. This process effectively avoids interference from low-weight or noisy features on the calculation results of high-weight features and helps retain the features most influential to model performance. The process flow diagram of this method is shown in the Figure 7.

### 2.3 INFO algorithm for optimizing parameter selection and enhancing model generalization

To reduce the impact of parameter combinations on model training performance, this study adopts the Weighted Mean of Vectors algorithm (INFO) (34), which aims to enhance the model’s generalization ability by optimizing its parameter configuration. The algorithm is based on the principle of weighted mean and updates the positions of the vectors through three key steps: update rules, vector combination, and local search. First, in the update rule stage, the algorithm combines the mean method with the convergence acceleration principle to generate new optimized vectors, thereby enhancing the stability and convergence speed of the search process. Next, in the vector combination stage, the optimized vectors obtained from the update rule are combined with the original vectors using a weighted approach, enhancing the information extension and development capability to improve global optimization performance. Finally, in the local search stage, the algorithm uses global position information and the weighted mean strategy to perform a local search, effectively avoiding the trap of local optima and ensuring that the search process moves toward the global optimal solution. This optimization algorithm not only enhances the model’s search capability in high-dimensional spaces but also improves its robustness and generalization ability in complex tasks.

### 2.4 SMOTE for addressing class imbalance in sleep stage data

To address the issue of class imbalance in the data samples due to the low proportion of the N1 stage during the sleep cycle, this study employs the Synthetic Minority Over-sampling Technique (SMOTE) (35) for feature augmentation. The advantages of the SMOTE feature augmentation method are as follows: First, it generates new synthetic sample points by interpolating between minority class samples rather than simply replicating existing minority class samples. Second, in the feature space, the method selects several nearest neighbors of a minority class sample and generates new samples via linear interpolation between the sample and these neighbors. The formula for SMOTE is defined in Equation (3):


(3) $$\mathrm{New}\;\text{Sample}=X_{i}+\lambda \times (X_{i}^{\mathrm{NN}}-X_{i})$$

where $X_{i}$ is the minority class sample, $X_{i}^{\mathrm{NN}}$ is its nearest neighbor, and λ is a random number between 0 and 1, representing the interpolation ratio. This approach increases the number of minority class samples while retaining the original distribution of the data, thus avoiding the overfitting thus avoiding the overfitting that may result from simply duplicating minority class samples.

### 2.5 Sleep staging based on fast-ABC boost

To address the problem of low accuracy in traditional classifiers, this study proposes a classifier modeling strategy based on Fast-ABC Boost (36). This model is an iterative algorithm that integrates a series of weak classifiers (regression trees) and selects appropriate base classes during the iteration process, achieving strong classification performance. Equation (4) defines the first derivative of the loss function with respect to the base class, while Equation (5) defines the second derivative.


(4) $$\frac{\partial L_{i}}{\partial F_{i,k}}=(r_{i,0}-p_{i,0})-(r_{i,k}-p_{i,k})$$

(5) $$\frac{\partial^{2}L_{i}}{\partial F_{i,k}^{2}}=p_{i,0}(1-p_{i,0})+p_{i,k}(1-p_{i,k})+2p_{i,0}p_{i,k}$$

Where $F_{i,k}$ represents the probability of the *i*th sample belonging to category k. $\frac{\partial L_{i}}{\partial F_{i,k}}\partial L_{i}/\partial F_{i,k}$ denotes the first derivative of the loss function $L_{i}$ with respect to the base class $F_{i,k}$ , providing gradient information about the loss function with respect to the base class. This guides the algorithm to adjust the direction under the current category probabilities to minimize the loss. $\frac{\partial^{2}L_{i}}{\partial F_{i,k}^{2}}\partial^{2}L_{i}/\partial F_{i,k}^{2}$ represents the second derivative of the loss function $L_{i}$ with respect to the base class $F_{i,k}F_{i,k}$ , providing curvature information of the loss function and helping to select an appropriate step size, accelerating the convergence of the algorithm. By integrating these derivatives, the base class selection algorithm can adjust the category probabilities in time during the iteration process to optimize model performance. This allows the algorithm to make more informed decisions during the tree splitting process, thus improving the robustness and accuracy of the model. Compared to the traditional ABCLogitBoost, which uses an “exhaustive search” strategy, the Fast-ABC Boost algorithm improves computational efficiency and enhances model robustness by introducing three new parameters for smarter and more efficient base class selection. In ABCLogitBoost, base class selection is performed through exhaustive search, where the algorithm tries each class as a base class and selects the one that minimizes the training loss. Fast-ABC Boost introduces the “s-worst class” strategy: During each iteration, the algorithm only selects the best-performing class from the s worst-performing classes (where 1 ≤ s ≤ K, and K is the total number of classes). Additionally, Fast-ABC Boost introduces the “gap” parameter g and the “warm-up” parameter w to optimize the frequency of base class selection. Base class selection begins after w iterations of warm-up, and then the search is performed once every g + 1 iterations. This reduces the frequency of base class selection, lowering computational costs, while maintaining model efficiency without significantly affecting classification performance. By appropriately setting the parameters s, g and w, Fast-ABC Boost can achieve better classification performance than the exhaustive strategy, demonstrating superior robustness and efficiency in multi-class tasks.

In this study, the dataset is randomly divided into a training set and a test set in an 8:2 ratio. The INFO algorithm is used for systematic tuning of the model’s hyperparameters. By testing each possible s-worst class option as the base class selection scheme, the aim is to minimize the impact of parameter combinations on model performance and generalization. Once the optimal hyperparameters are determined, they are fixed and used in all subsequent experiments. To obtain a robust and realistic estimate of model performance, we further conduct five-fold cross-validation across the entire dataset using the selected parameter configuration. Final performance metrics are reported as the average across all five folds.

## 3 Experiment

### 3.1 Dataset description

The BCG data utilized in this study were provided by Bobo Technology (Suzhou) Co., Ltd., in collaboration with the affiliated Sir Run Run Shaw Hospital. The project received ethical approval from the Ethics Committee of Sir Run Run Shaw Hospital, Zhejiang University School of Medicine (Approval No. 20190520-67). The raw signal data were collected from 10 independent recordings using a BCG-based sleep monitoring device (Model: MD-EA, Medical Device Registration No. 20232070772). Each recording captured a full night of sleep from a different subject, with an average wake time of 1.02 ± 0.78 h, an average sleep time of 7.84 ± 1.43 h, and an average total monitoring duration of 8.86 ± 0.74 h. The study population consisted of 10 healthy individuals (3 males and 7 females), with an average age of 28 years. All participants were in good physical condition with no known health issues. All data were manually calibrated by three licensed physicians from Sir Run Run Shaw Hospital, affiliated with Zhejiang University’s Medical College. Sleep stages were annotated in 30-s epochs. Any segments with inconsistent annotations among the three physicians were considered invalid and subsequently excluded from the dataset. After this rigorous validation process, a total of 10,201 valid sample data points were retained for analysis. Details of the dataset parameters are summarized in Table 1.

**Table 1:** Sleep sample data statistics.

Number category	Value
WK	1,082 (10.61%)
REM	2,183 (21.40%)
NREM (N1)	442 (04.33%)
NREM (N2)	4,414 (43.27%)
NREM (N3)	2080 (20.39%)
Total	10,201 (100%)

### 3.2 Evaluation index

To evaluate the performance of the models in the sleep staging task, this study employed accuracy (ACC), precision (PRE), recall, F1-score, and Cohen’s kappa as evaluation metrics. The calculation methods for these metrics are presented in Equations (6–12).


(6) $$\text{Accuracy}=\frac{\mathrm{TP}+\mathrm{TN}}{\mathrm{TP}+\mathrm{FP}+\mathrm{TN}+\mathrm{FN}}$$

(7) $$\text{Precision}=\frac{\mathrm{TP}}{\mathrm{TP}+\mathrm{FP}}$$

(8) $$F1=\frac{2\mathrm{TP}}{2\mathrm{TP}+\mathrm{FP}+\mathrm{FN}}$$

(9) $$\text{Recall}=\frac{\mathrm{TP}}{\mathrm{TP}+\mathrm{FN}}$$

(10) $$\text{Kappa}=\frac{p_{0}-p_{e}}{1-p_{e}}$$

(11) $$p_{0}=\frac{\mathrm{TP}+\mathrm{TN}}{\mathrm{TP}+\mathrm{FP}+\mathrm{TN}+\mathrm{FN}}$$

(12) $$p_{e}=\frac{\left(\mathrm{TP}+\mathrm{FN})(\mathrm{TP}+\mathrm{FP})+(\mathrm{TN}+\mathrm{FP})(\mathrm{TN}+\mathrm{FN}\right)}{{\left(\mathrm{TP}+\mathrm{FP}+\mathrm{TN}+\mathrm{FN}\right)}^{2}}$$

Where True Positive (TP) refers to the number of samples that are correctly classified as category *i*. False Negative (FN) refers to the number of category *i* samples that are misclassified as not belonging to category *i*. False Positive (FP) refers to the number of non-category *i* samples that are misclassified as belonging to category *i*. True Negative (TN) refers to the number of samples correctly identified as not belonging to category *i*.

## 4 Results

### 4.1 Feature selection

**Figure 8:**
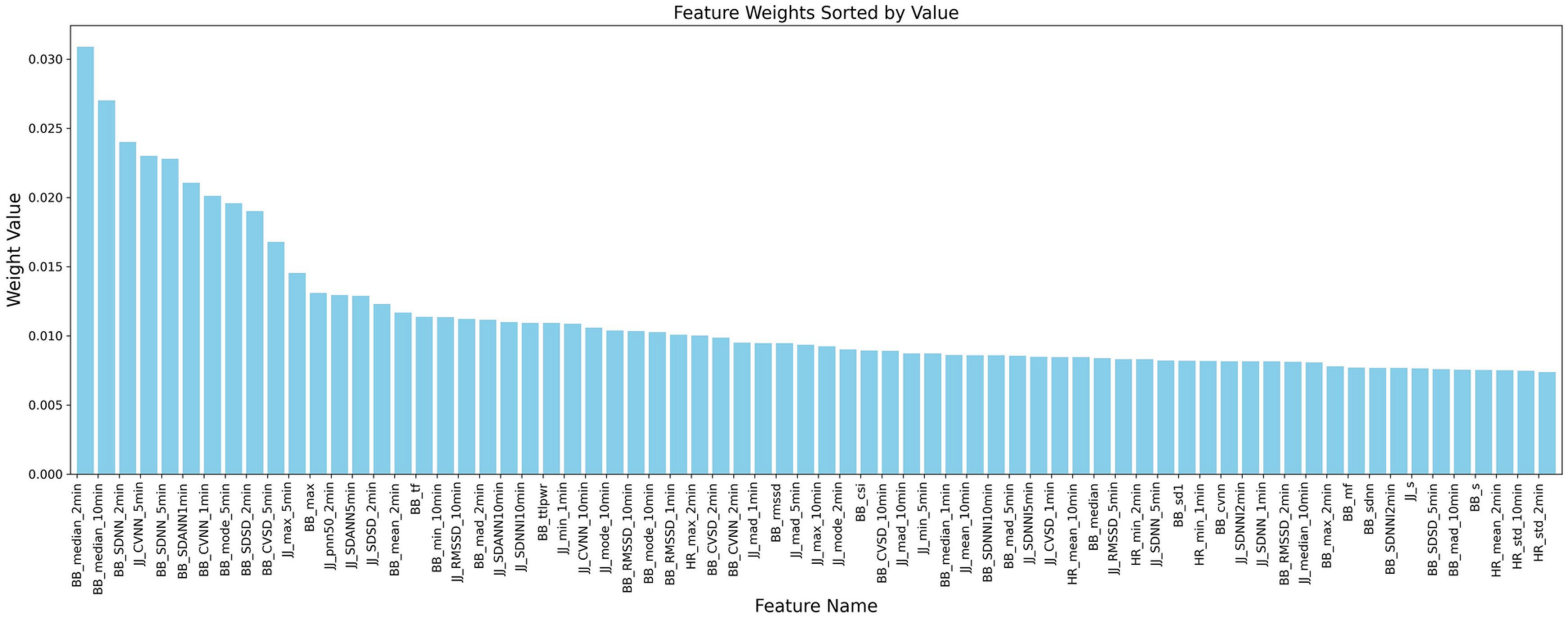
Analysis of sleep stage feature weights.

This study is based on the first-order difference of the peak position sequences of heart rate and respiratory signals in 30-s and longer time segments. A feature extraction method based on the JJ_i_ and BB_i_ sequences is designed to explore their application in the analysis of heart rate variability (HRV) and respiratory rate variability (RRV) feature sequences. We comprehensively considered various HRV and RRV features, including time-domain, frequency-domain, non-linear domain, complexity index, Poincaré plot geometry, and long-time window time-domain features. These features can more comprehensively characterize the complex variations in physiological signals and reflect the long-term trends of sleep. A total of 232 HRV and RRV features were extracted, as detailed in Table 2. To further select HRV and RRV features that are strongly correlated with different sleep stages, feature selection was conducted using an attention mechanism combined with the XGBoost algorithm. The parameters were determined by the INFO algorithm, with the following settings: (Number of iterations: 3, Weight ratio: 0.796). Ultimately, 70 features were selected, and their weights are shown in Figure 8.

**Table 2:** HRV and RRV feature statistics.

Count	Feature
**34**	**Time domain features**
10	Mean, Median, Max, Min, and MAD for the JJ sequence and BB sequence.
12	RMSSD, SDNN, SDSD, CVNN, CVSD, and RMSA for the JJ sequence and BB sequence.
4	NN20, PNN20, NN50, and PNN50 for the JJ sequence.
4	Mean, Min, Max, and Std for the HR sequence.
4	TINN and HTI for the JJ sequence and BB sequence.
**44**	**Frequency domain features**
16	LF, HF, MF, VL, TF, TLF, ULF, and Ttlpwr for the JJ sequence and BB sequence.
10	LFf, HFf, MFf, TLFf, and TFf for the JJ sequence and BB sequence.
8	LFn, HFn, MFn, and TLFn for the JJ sequence and BB sequence.
6	LFHF, MFLF, and TLFLF for the JJ sequence and BB sequence.
4	HFmaxf and HFamp for the JJ sequence and BB sequence.
**14**	**Characteristics of the poincaré plot geometry**
8	SD1, SD2, SD1SD2, and S for the JJ sequence and BB sequence.
6	CVI, CSI, and CSI_Modified for the JJ sequence and BB sequence.
**14**	**Indices of heart rate asymmetry**
14	GI, C1d, C1a, SD1d, SD1a, C2d, C2a, SD2d, SD2a, SD2I, Cd, Ca, SDNNd, and SDNNa for the JJ sequence.
**2**	**Indices of complexity**
2	SampEn for the JJ sequence and BB sequence.
**124**	**Time series characteristics under multi-scale time windows**
48	Mean, Median, Mode, Max, Min, and MAD for the JJ sequenceand BB sequence in the i-minute time window (i = 1, 2, 5, 10).
56	RMSSD, SDNN, SDSD, CVNN, CVSD, SDANN, SDNNI for the JJ sequence and BB sequence in the i-minute time window (i = 1, 2, 5, 10).
4	PNN50 for the JJ sequence in the i-minute time window (i = 1, 2, 5, 10).
16	Mean, Min, Max, and Std for the HR sequence in the i-minute time window (i = 1, 2, 5, 10).

### 4.2 Model performance evaluation

The parameters of the Fast-ABC Boost model were obtained using the INFO algorithm during hyperparameter tuning on the initial 8:2 split. To ensure consistency and minimize the influence of parameter combinations, the selected configuration is fixed and applied to all subsequent experiments. The final hyperparameter settings are as follows: (Learning rate: 0.4546, Maximum number of iterations: 645, Search: 3, Gap: 19, Warm-up: 34). To provide a robust and generalizable evaluation, all performance metrics reported below were obtained through five-fold cross-validation conducted on the entire dataset. The experimental groups are set as follows:

- Experiment 1: Validation and evaluation of the datasets using HRV and RRV features, with results shown in Table 3.
- Experiment 2: Validation and evaluation of the datasets using HRV and RRV features within long time windows, with results shown in Table 4.
- Experiment 3: Validation and evaluation of the datasets using HRV and RRV features within long time windows, followed by dimensionality reduction using the feature selection framework proposed in this study, with results shown in Table 5.
- Experiment 4: Validation and evaluation of the datasets using HRV and RRV features within long time windows, followed by dimensionality reduction using the feature selection model and mainstream linear and nonlinear dimensionality reduction models, as shown in Table 6.

**Table 3:** Sleep staging results using HRV and RRV features.

Confusion matrix	Sleep stage performance			Overall performance	
		N1	N2			N3	REM	W	Recall	PRE	F1
N1	11	249	35	95	52	02.49%	11.00%	04.06%	ACC	57.90%
N2	39	3,361	459	424	131	76.14%	60.42%	67.37%	PRE	44.21%
N3	9	909	972	138	52	46.73%	57.72%	51.65%	Recall	48.12%
REM	29	788	149	1,046	171	47.92%	54.14%	50.84%	F1	47.25%
W	12	256	69	229	516	47.69%	55.97%	51.50%	Kappa	50.01%

**Table 4:** Sleep staging results using HRV and RRV features from long time windows.

Confusion matrix	Sleep stage performance			Overall performance	
		N1	N2			N3	REM	W	Recall	PRE	F1
N1	50	287	18	50	37	11.31%	23.04%	15.17%	ACC	84.02%
N2	87	3,983	157	118	69	90.24%	82.60%	86.25%	PRE	70.30%
N3	11	279	1735	32	23	83.41%	89.29%	86.25%	Recall	73.47%
REM	26	127	11	1987	32	91.02%	88.63%	89.81%	F1	71.34%
W	43	146	22	55	816	75.42%	83.52%	79.26%	Kappa	80.74%

**Table 5:** Sleep staging results using dimensionality-reduced HRV and RRV features from long time windows.

Confusion matrix	Sleep stage performance			Overall performance	
		N1	N2			N3	REM	W	Recall	PRE	F1
N1	116	252	3	34	37	26.24%	49.36%	34.27%	ACC	89.85%
N2	63	4,143	111	61	36	93.86%	87.87%	90.77%	PRE	78.15%
N3	1	165	1901	6	7	91.39%	93.74%	92.55%	Recall	83.25%
REM	9	53	0	2,115	6	96.89%	94.17%	95.51%	F1	79.88%
W	46	102	13	30	891	82.35%	91.20%	86.55%	Kappa	87.74%

**Table 6:** Performance evaluation of feature selection model.

Method	Evaluation index				
	ACC	PRE	Recall	F1	Kappa
PCA	59.03%	43.83%	52.25%	45.81%	52.27%
ICA	59.19%	43.64%	52.26%	45.61%	52.19%
LDA	62.07%	57.04%	58.43%	57.41%	54.35%
t-SNE	49.76%	40.58%	42.13%	41.10%	39.23%
Autoencoder	72.06%	58.90%	63.11%	60.25%	66.43%
LLE	73.92%	62.02%	66.24%	63.61%	68.61%
MDS	68.96%	55.04%	60.92%	56.86%	62.95%
Lasso	86.15%	72.72%	78.12%	74.01%	83.31%
RFE	86.70%	75.45%	78.62%	76.62%	83.90%
ReliefF	85.27%	75.02%	77.47%	75.87%	82.07%
XGBoost	87.05%	74.56%	79.88%	76.19%	84.39%
Ours	89.85%	78.15%	83.25%	79.88%	87.74%

## 5 Discussion

This study establishes the mapping relationship between BCG signal data and sleep stages by analyzing and extracting high-dimensional HRV and RRV features from the BCG signals, thereby linking the signal data to the corresponding sleep stages. Additionally, this study introduces long-time window features to better capture the long-term trends in sleep. Through the feature selection framework that combines the attention mechanism and XGBoost, the majority of the selected features are long-time window features. This underscores the importance of feature extraction using longer time window, which can capture the dynamic changes in biological information and better represent the sequential relationship of sleep as a continuous event, thus deepening the understanding of sleep staging. In experiments using high-dimensional HRV and RRV features, an accuracy of 57.90% was achieved. When incorporating long-time window features, the accuracy increased to 84.02%, representing a 26.12% improvement compared to using only 30-s window features. This highlights the critical role of long-time window features in enhancing sleep staging performance.

Moreover, different feature combinations can influence the performance of the classifier in sleep staging. In experiments comparing HRV and RRV features before and after dimensionality reduction, the dimensionality-reduced dataset achieved an accuracy 5.83% higher than the original, non-reduced dataset. This indicates the presence of redundant and noisy features in the high-dimensional data, which can negatively affect model performance and generalization ability, thus demonstrating the necessity of feature selection.

To rigorously evaluate the effectiveness of our proposed attention-based XGBoost feature selection framework, we first conducted a comprehensive evaluation using a variety of mainstream feature selection and dimensionality reduction techniques, as summarized in Table 6. Among traditional linear methods, PCA and ICA achieved classification accuracies of 59.03 and 59.19%, respectively. LDA performed slightly better at 62.07% due to its supervised nature. However, these methods showed limited performance, likely because they rely on global projections that are not robust to irrelevant or noisy features commonly present in high-dimensional HRV and RRV data. Similar limitations were observed in nonlinear dimensionality reduction methods. For example, t-SNE produced the lowest accuracy (49.76%) among all methods. This is expected, as t-SNE is primarily designed for visualization purposes rather than classification—it prioritizes preserving local structure while potentially distorting global relationships. In contrast, other nonlinear methods such as Autoencoder (72.06%), LLE (73.92%), and MDS (68.96%) performed better, although still inferior to feature selection-based approaches. These methods can capture nonlinear patterns to some extent, but often fail to eliminate irrelevant or redundant features, leading to suboptimal classification outcomes. Feature selection methods, including Lasso (86.15%), RFE (86.70%), ReliefF (85.27%), and XGBoost (87.05%), explicitly rank and retain only the most informative features, avoiding the dilution of useful signals. These methods have demonstrated consistently better performance, highlighting the importance of targeted feature filtering over general-purpose dimensionality reduction in noisy physiological datasets.

The proposed method achieved the best overall performance, with an accuracy of 89.85%, outperforming all baseline methods. A key innovation lies in the integration of an attention mechanism with the XGBoost algorithm for feature selection. Specifically, the attention mechanism assigns adaptive weights to each high-dimensional HRV and RRV feature, emphasizing features more relevant to the sleep stage classification task. These weighted features are then input into the XGBoost model, which performs feature selection based on its tree-based importance metrics. Unlike conventional one-pass approaches, the propose method incorporates a multi-round iterative refinement mechanism, wherein attention-based weighting and XGBoost-based ranking are repeatedly updated. This iterative strategy enables progressive suppression of irrelevant or redundant features while reinforcing the influence of informative attributes. These design choices make our method highly robust to the challenges inherent in BCG-derived HRV and RRV features, which are often high-dimensional and noisy. As a result, the proposed framework is particularly effective in isolating features that are strongly correlated with transitions between sleep stages, thus improving not only classification accuracy but also the interpretability and reliability of physiological signal analysis.

## 6 Conclusion

This paper proposes a feature dimensionality reduction method based on BCG sleep staging technology to enhance the accuracy of sleep staging. High-dimensional HRV and RRV features extracted from BCG signals are used in conjunction with an attention mechanism and the XGBoost algorithm to select appropriate feature combinations for sleep staging research. Since BCG signals are an indirect recording of cardiac activity, they are subject to noise interference from the external environment and body movements. Therefore, this study employs low-pass filtering and continuous wavelet transform, automatically selecting the appropriate frequency bands in a multi-scale parameter space to separate and reconstruct the respiratory and heart rate signals from the original signal. This process extracts peak information, forming the JJ_i_ and BB_i_ sequences.

This study presents a novel feature selection framework that effectively bridges deep learning and ensemble learning strategies for physiological signal analysis. By integrating attention-based dynamic weighting with XGBoost and incorporating an iterative refinement mechanism, the framework demonstrates strong robustness to noise and redundancy—common challenges in high-dimensional BCG-derived HRV and RRV features. This approach successfully improves sleep stage classification performance.

To address the class imbalance in the dataset, SMOTE is applied to enhance the minority class samples, allowing the model to focus more on these underrepresented classes. Finally, the Fast-ABC Boost model is employed for sleep staging. Experimental results show that the feature set selected by the proposed feature selection framework, when applied to the Fast-ABC Boost model, achieves an accuracy of 89.85%, outperforming mainstream feature dimensionality reduction models in terms of classification performance.

This study utilizes BCG signals for sleep staging. Compared to traditional EEG, EOG, and ECG signals, BCG signals offer the advantage of non-contact measurement, providing richer biological feedback. This sleep staging method holds significant potential for broad application, particularly in home and mobile environments, offering a convenient solution for the auxiliary diagnosis and treatment of sleep disorders. However, due to the current lack of publicly available standard datasets for BCG-based sleep staging, this study relies on a limited dataset annotated by experts. The small sample size constrains the generalizability of the results. Future efforts will focus on constructing large-scale, high-quality datasets to enhance model robustness and promote progress in sleep health technologies.

## Data Availability

Due to the nature of the data (potentially sensitive and identifying), the Ethics Committee of Sir Run Run Shaw Hospital, Zhejiang University School of Medicine did not approve public access to the data. Requests to access the datasets should be directed to BL, bantl@zjsru.edu.cn.
